# Detection of *Giardia duodenalis* Assemblages A and B in Human Feces by Simple, Assemblage-Specific PCR Assays

**DOI:** 10.1371/journal.pntd.0001776

**Published:** 2012-08-28

**Authors:** Ilaria Vanni, Simone Mario Cacciò, Lindy van Lith, Marianne Lebbad, Staffan G. Svärd, Edoardo Pozio, Fabio Tosini

**Affiliations:** 1 Department of Infectious, Parasitic and Immunomediated Diseases, Istituto Superiore di Sanità, Rome, Italy; 2 Faculty of Veterinary Medicine, University of Utrecht, Utrecht, The Netherlands; 3 Department of Diagnostics and Vaccinology, Swedish Institute for Communicable Disease Control (SMI), Solna, Sweden; 4 Department of Cell and Molecular Biology, BMC, Uppsala University, Uppsala, Sweden; University of Vermont, United States of America

## Abstract

The flagellated protozoan *Giardia duodenalis* is a common gastrointestinal parasite of mammals, including humans. Molecular characterizations have shown the existence of eight genetic groups (or assemblages) in the *G. duodenalis* species complex. Human infections are caused by assemblages A and B, which infect other mammals as well. Whether transmission routes, animal reservoirs and associations with specific symptoms differ for assemblage A and assemblage B is not clear. Furthermore, the occurrence and clinical significance of mixed (A+B) infections is also poorly understood. To date, the majority of PCR assays has been developed to identify all *G. duodenalis* assemblages based on the use of primers that bind to conserved regions, yet a reliable identification of specific assemblages is better achieved by *ad hoc* methods. The aim of this work was to design simple PCR assays that, based on the use of assemblage-specific primers, produce diagnostic bands of different lengths for assemblage A and B. We first generated novel sequence information from assemblage B, identified homologous sequences in the assemblage A genome, and designed primers at six independent loci. Experiments performed on DNA extracted from axenic cultures showed that two of the six assays can detect the equivalent of a single cyst and are not negatively influenced by disproportions between DNA of each assemblage, at least up to a 9∶1 ratio. Further experiments on DNAs extracted from feces showed that the two assays can detect both assemblages in single tube reactions with excellent reliability. Finally, the robustness of these assays was demonstrated by testing a large collection of human isolates previously typed by multi-locus genotyping.

## Author Summary

*Giardia duodenalis* is an important cause of diarrhoea in humans worldwide, even if the burden of infection is higher in developing countries where the poor sanitary conditions favour the contamination of water and food with infective cysts. The parasite is considered as a species complex that comprises at least eight distinct genetic groups, referred to as assemblage A to H. Humans are infected only by assemblages A and B, which can only be distinguished by molecular methods. The clinical manifestations of giardiasis in humans are highly variable and range from the absence of symptoms to acute or chronic diarrhoea. Since genetic traits that influence the virulence are yet unknown, the identification of the assemblage is considered a primary element in the study of human giardiasis. Current methods are time consuming and/or require expensive instruments. Here, we describe the development and application of single step PCR methods that allow to detect and distinguish assemblages A and B from human fecal specimens simply by gel electrophoresis of the amplification products. The novelty of the assays described in our manuscript is the reliability in detecting mixed infections and the applicability of the methodology in laboratories with basic molecular equipment.

## Introduction

*Giardia duodenalis* is an important cause of diarrhea in humans worldwide and is responsible for an estimated 2.8×10^8^ cases per year [1]. The infection is transmitted by the fecal-oral route through ingestion of infective cysts. The prevalence of the infection is higher in developing countries as the poor sanitary conditions favour the contamination of water and food with cysts. Approximately 200 million people have symptomatic giardiasis in Asia, Africa and Latin America and about 500,000 new cases are reported each year [2]. The parasite has been included in the Neglected Diseases Initiative of the World Health Organization (WHO) due to its diffusion among children in these regions of the world [3].

A considerable amount of data has shown that *Giardia duodenalis* should be considered as a species complex that comprises at least eight distinct genetic groups, referred to as assemblage A to H [4]. Isolates from the different assemblages show little, if any, morphologic variation, thus their identification is currently based on molecular characterization. To date, only assemblages A and B have been associated with human infections, but are also found in a number of other mammalian hosts [5].

The clinical manifestations of giardiasis in humans are highly variable and range from the absence of symptoms to acute or chronic diarrhoea often associated with dehydration, weight loss, abdominal pain, nausea and vomiting [6]. The severity of disease is likely determined by the interplay between the host status (e.g., age, nutritional and immunological conditions) and intrinsic features of the parasite (e.g., assemblage and genotype). However, genetic traits that influence the virulence and other aspects of the infection are unknown and efforts to correlate the parasite genetic make-up and the clinical symptoms in the host have generated controversial results [7]. It is also conceivable that the two assemblages have different epidemiological features as infectivity, zoonotic potential and route of transmission, but definite differences are not yet established.

Until recently assemblages A and B have been considered as genetic variants of the same species. Nowadays, the bulk of the genetic data indicates that the two assemblages A and B are probably two independent species, despite the morphological similarity. The genome of the assemblage A (strain WB) has been sequenced [8] and, more recently, a draft of the genome of the assemblage B (strain GS) has been published [9]. The comparison of the genomes, which show only the 77% of identity at the nucleotide level, supports the hypothesis that assemblage A and B represent different species [9]. Nevertheless, the taxonomy of the *G. duodenalis* species complex remains a matter of discussion [4], [10].

The aim of this work is the development of PCR assays that, through the use of assemblage-specific primers generating amplification products of different size, allow the detection of assemblage A and B in human fecal samples.

## Methods

### Ethics statement

The human samples from Italy have been collected by various Italian health services and have been received at ISS on different times for a confirmation of the diagnosis of giardiasis. All samples were received in an anonymous form. The human samples from Sweden are from a study approved by the Regional Ethics Committee of Karolinska Institutet, Stockholm, Sweden. Written consent for being part of the study was obtained from each patient and parents or primary caretakers signed for their children.

### General microbiological and DNA techniques

The recombinant and microbiological techniques, media, the preparation of plasmid DNA and the isolation of restricted fragments all followed standard procedures [11]. DNA sequencing was performed using Sanger's procedure [12].

### Construction of a genomic library for the selection of assemblage B specific clones

The *G. duodenalis* isolate Ad-28 [13] has been used as a source of assemblage B genomic DNA. 1 µg of DNA was digested with BstYI (New England Biolabs) and ligated using the Quick ligation kit (New England Biolabs) to the plasmid pBLUESCRIPT, previously linearized using *Bam* HI (New England Biolabs). *Escherichia coli* XL1-Blue competent cells were used as a recipient strain and transformed with 1.2 µg of ligated DNA and plated on LB agar plates containing 1 mM IPTG, 30 µg/ml X-Gal and 100 µg/ml ampicillin to select the white recombinant colonies. Approximately 100 recombinant clones from this library were randomly chosen and replicated from the original plates on 3 square plates with grid (6×6 cells), and each clone was named with the coordinates of its position. From each clone plasmid DNA was prepared using Qiaprep miniprep (Qiagen) following the manufacturer's protocol. Plasmidic DNAs from recombinant colonies were used to amplify and sequence the exogenous inserts using M13-for (CGCCAGGGTTTTCCCAGTCACGAC) and M13-rev (GTCATAGCTGTTTCCTGTGTGA) vector-specific primers using GoTaq Green Master Mix (Promega) under these conditions: 2 min at 94°C, 35× (30 sec at 94°C, 30 sec at 62°C, 1 min at 72°C), 7 min at 72°C. Amplification products were analyzed on 1.5% agarose gel stained with ethidium bromide and suitable fragments (200–500 bp in length) were purified with Qiaquick PCR purification kit (Qiagen) and sequenced.

### Bionformatic analysis

Sequences of recombinant inserts were assembled using the software Seqman II (DNASTAR). The homologous genes in the assemblage A genome were identified by BLAST both at http://blast.ncbi.nlm.nih.gov/Blast.cgi and at www.giardiadb.org. Alignments were obtained using CLUSTAL W [14].

### PCR experiments

Assemblage-specific PCR assays were performed on a Veriti 96 Well Thermal-cycler (Applied Biosystem) using the GoTaq Green Master Mix (Promega) or the HotStarTaq Master Mix (Qiagen). Concentration of primers in PCR reactions was 10 pM each and sequences of primer pairs are reported in Table 1. All reactions started with an activation-denaturation step at 94°C for 5 min and then were carried out for 40 cycles, each consisting of 94°C for 30 sec, an annealing step of 30 sec at a temperature that varied depending on the marker (see below), and of 72°C for 30 sec. A final extension step was carried out at 72°C for 7 min. Optimal annealing temperatures were: 52°C for 3B4-HP, 56°C for 4E1-HP and 5C1-P21 , 58°C for 1A3-HCP, 60°C for 4F1-HP and 5A2-VSP. PCR products were electrophoresed on ethidium bromide-stained 2% agarose gels in TAE 1× at 60 Volt for 90 min and photographed with Geliance 600 imaging system (Perkin Elmer). The DNA molecular weight marker XIII (Roche) was used as size standard in gel electrophoresis.

**Table 1:** List of primers for the assemblage-specific PCRs and sizes of the amplicons.

Assay	Assemblage	Primer	PCR product (bp)
**1A3-HCP**	A	For TGCTTCGGGGCGCATGCA	326
		Rev CAGGTAGCAGAGAATCCCTCG	
	B	For ATGTGTCAGTGTGACAGTAACGT	347
		Rev GTGACTGTGCCGTTGAGGCAGT	
**3B4-HP**	A	For CTGGTTTGAACTTTACTTCCAG	169
		Rev GATGGTGCTGACAGCGTTG	
	B	For CTCCGGCCTTTGGTACACGA	249
		Rev TCCTCAAATCGCTGTCCCT	
**4E1-HP**	A	For AAAGAGATAGTTCGCGATGTC	165
		Rev ATTAACAAACAGGGAGACGTATG	
	B	For GAAGTCATCTCTGGGGCAAG	272
		Rev GAAGTCTAGATAAACGTGTCGG	
**4F1-HP**	A	For GTGCGTGAGTCCTTGGCTG	229
		Rev GGCCGCCTCGTCTTCTATG	
	B	For AGAGGCAGAGGTGCAACTTG	180
		Rev GTCTCAGCGTTAGCGATTGC	
**5A2-VSP**	A	For TGCAGACCCAAAAACAGGCC	187
		Rev ACTTGTGCACTCTGCCTCCG	
	B	For CCTGTGTAGAAGCTAGTGGG	272
		Rev ATTACACAGAAGACACTTCTTCCC	
**5C1-P21**	A	For ATGCTAGCCGTAGTTAATAAGG	303
		Rev ACCGGCCTTATCTACCAGC	
	B	For TTAATAGAAATGCTTTCGACACG	249
		Rev TTGCTACAGCAGAAAGGTGC	

### Human clinical samples

The panel of 15 human samples from Italy has been previously described [15] as well as the panel of 51 human samples from Sweden [16]. All those samples have been genotyped at the level of assemblage using a multi-locus typing scheme based on the sequence analysis of the beta-giardin (ß-giardin), triose phosphate isomerise (TPI) and glutamate dehydrogenase (GDH) genes.

## Results

### Construction of a genomic library of *G. duodenalis* Ad-28 strain and selection of clones

To identify novel sequences of *G. duodenalis* assemblage B, 29 randomly cloned fragments from a plasmid library of the Ad-28 strain were fully sequenced. The comparison of these sequences with the genome of assemblage A identified sequences with high levels of homology, most likely representing true orthologs. The genomic localization and the relative annotation of these sequences, as found in the GiardiaDB, including the assemblage E homologs [17], are given in the Table S1. A high level of inter-assemblage variability was found when comparing the homologous sequences of WB and GS strains, with identity values at the DNA level between 63 and 81%. Noteworthy, all the clone sequences corresponded to coding sequences, even if two of them (1A3 and 3B4) were annotated only in the assemblage A genome but not in the assemblage B draft genome (Table S1).

### Design and test of assemblage-specific PCR assays

For the design of PCR assays, a further selection of sequences was based on the following criteria: i) sequences showing full specificity for *G. duodenalis*; ii) unambiguous identification of the orthologs in assemblage A; iii) sufficient variability to design specific primer pairs for each assemblage. Six sequences were selected for further analysis following these criteria (Table 2).

**Table 2:** Selected clones from the library of *Giardia duodenalis* Ad-28 strain and their genome localizations on both assemblages B (GS) and A (WB).

Clone length	GS genomic sequence Contig: position (bp)	Annotation in GS Database	WB genomic sequence Gene ID	Identity GS/WB
1A3 882 bp	ACGJ01002644:214–557	None	GL50803_113531 High cysteine membrane protein EGF-like	76%
3B4 816 bp	ACGJ01000325:96–344	GL50581_2493 Hypothetical protein	GL50803_16690 Hypothetical protein	68%
4E1 336 bp	ACGJ01002726:53416–53687	GL50581_3242 Hypothetical protein	GL50803_13988 Hypothetical protein	81%
4F1 322 bp	ACGJ01002463:916–1095	None	GL50803_95908 Hypothetical protein	78%
5A2 313 bp	ACGJ01000140:326–473	None	GL50803_137610 VSP protein*	63%
5C1 350 bp	ACGJ01000922:40352–40600	GL50581_725: Protein 21.1	GL50803_15306 Protein 21.1	78%

* Orthologous gene identified on the basis of the similarity of the encoded peptide.

**Figure 1:**
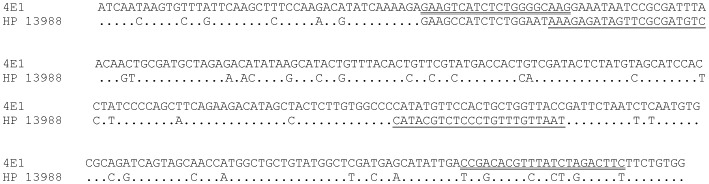
Primer design for the 4E1-HP assay. Alignment of the sequence of the 4E1 clone (assemblage B) with the homologous sequence of HP 13988 (assemblage A). Dots correspond to identical nucleotides. The primers designed for assemblage A amplification are underlined with single lines, whereas the primers designed for assemblage B amplification are underlined with double lines.

Primers were designed to amplify fragments of different size from each assemblage at each locus, thus allowing the identification of each assemblage after electrophoretic separation of PCR products (Table 1). An example of primer design for the 4E1/HP marker is shown in Figure 1, whereas information for the remaining markers is given in Table S2.

**Figure 2:**
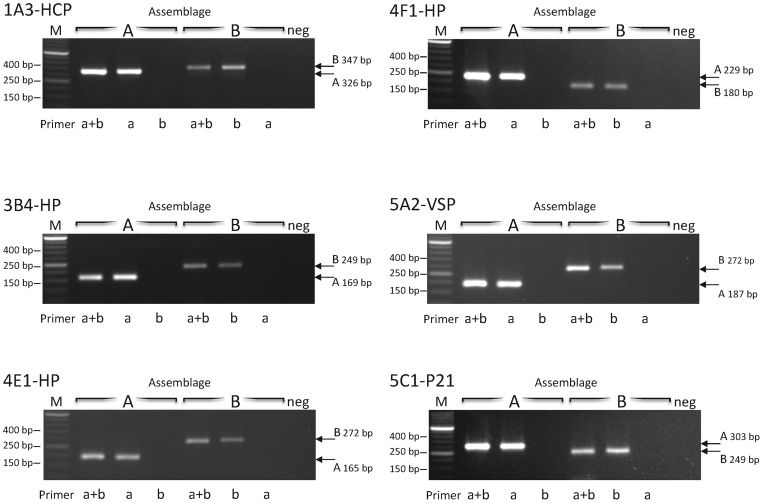
PCR assays at six independent genetic loci. Locus names are shown at the upper left corner of each panel. Capital letters in the upper lane indicate the reference DNA in each sample: A for assemblage A, B for assemblage B. Neg indicates the negative control (no DNA). Lower cases in the lower lane indicate the specific primer pair used for each reaction: a for the assemblage A specific pair; b for the assemblage B specific pair; a+b for the two primer pairs combined. Sizes of the assemblage-specific bands are indicated on the right. Size markers are indicated on the left.

The amplicons produced by these PCR assays on the reference DNA of assemblage A and B are shown in Figure 2 (throughout the manuscript, PCR assays are named combining the name of the clone and the acronym of gene name as reported in GiardiaDB, for example: 4E1-HP). All assays produced the expected bands only when the specific template and primers were used, and no amplification was observed when assemblage B specific primers were tested on assemblage A template or when assemblage A specific primers were tested on assemblage B template (see Figure 2).

### Assemblage-specific PCR assays on DNAs from human clinical specimens

**Figure 3:**
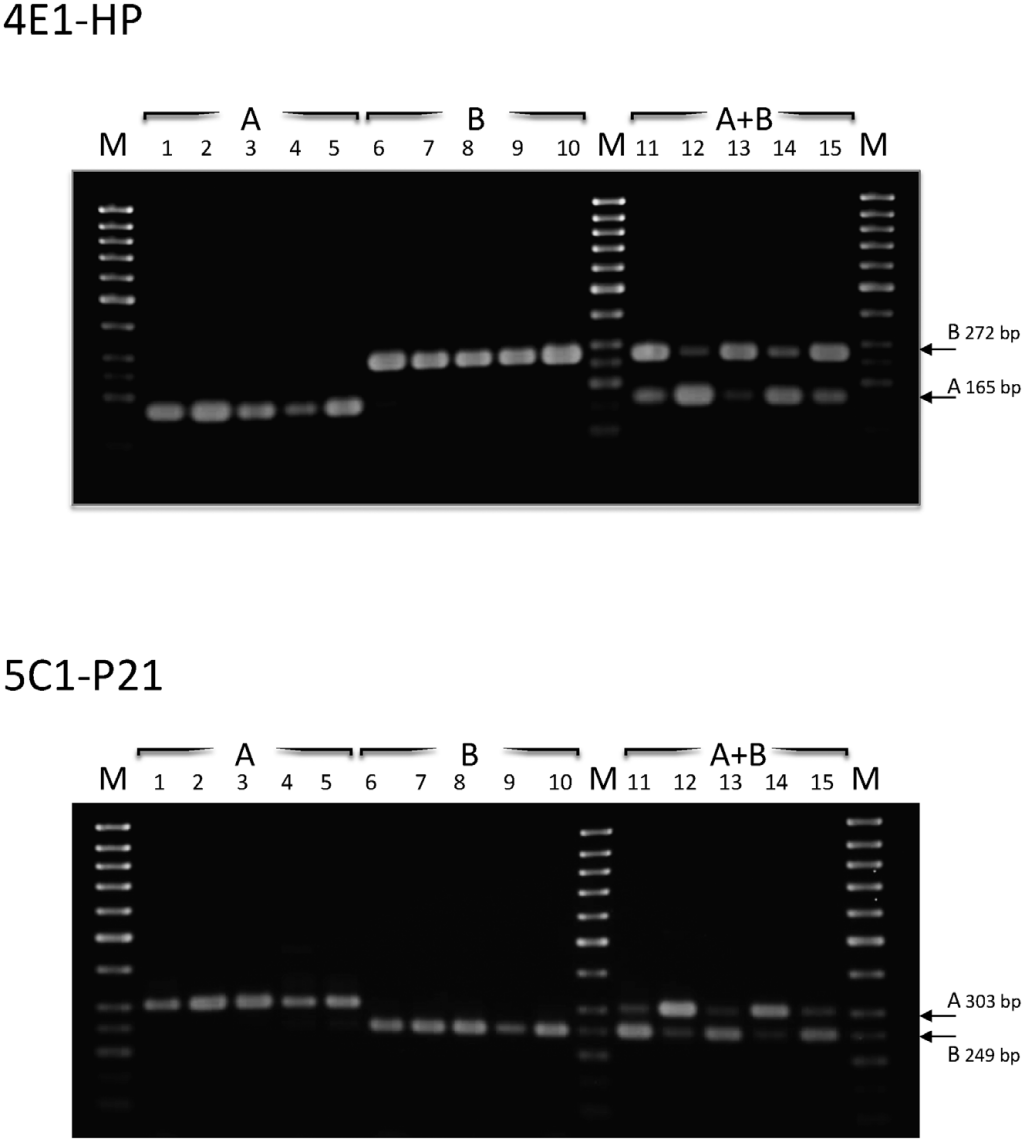
PCR amplification of the 4E1-HP and 5C1-P21 markers from 15 human fecal samples positive for *G. duodenalis*. Samples 1–5: assemblage A; samples 6–10: assemblage B; samples 11–15: mixed assemblage A+B.

To select the most appropriate assays for use on clinical isolates, 15 DNA isolates extracted from human fecal samples, previously genotyped as assemblage A (5 isolates), assemblage B (5 isolates) and mixed A+B assemblages (5 isolates), were tested using the six assays. To select for reliable single-step PCR assays, the reactions were run in the presence of primers for the two assemblages in the same tube. Three assays (4F1-HP, 3B4-HP and 5A2-VSP) yielded the expected bands on DNAs from either assemblage A or B when tested separately, but the interpretation of results was more difficult in case of mixed A+B infections. This was probably due to the different sensitivity of these assays in the amplification of one of the two templates (data not shown). The 1A3-HCP assay produced bands of similar size from each assemblage (i. e. 326 bp for A assemblage and 347 bp for B assemblage), therefore requiring a high resolution gel to detect mixed infections (data not shown). However, the 4E1-HP and 5C1-P21 assays, under these conditions, yielded excellent results in terms of sensitivity for both assemblages and were in total agreement with the genotyping results obtained previously for these isolates (Figure 3).

### Sensitivity of 4E1-HP and 5C1-P21 PCR assays and reliability on mixed samples

**Figure 4:**
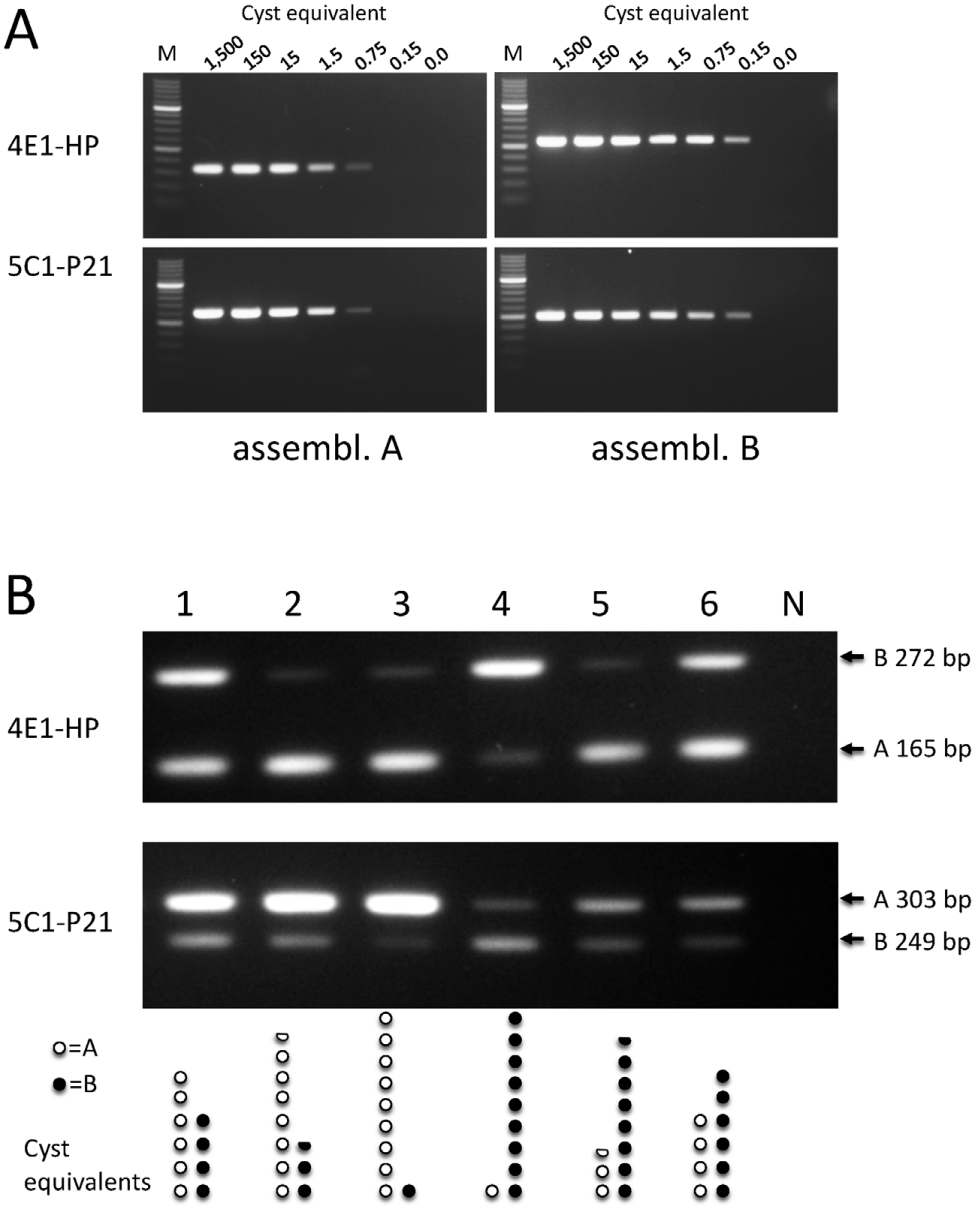
Sensitivity of 4E1-HP and 5C1-P21 PCR assays. Panel A: sensitivity test on serial dilutions of reference DNAs (WB for assemblage A and Ad28 for assemblage B). Left images show results obtained with assemblage A; right images show results obtained with assemblage B. The amount of DNA template, reported as the number of cysts equivalent, is shown at the top of the images. Panel B: results of assays tested on different proportions of the same reference DNAs. The following A to B ratios were tested: 6∶4 (1); 7.5∶2.5 (2,); 9∶1 (3); 1∶9 (4); 2.5∶7.5 (5); and 4∶6 (6). N indicates the negative control.

To obtain a more accurate evaluation of the sensitivity, serial dilutions of reference DNAs from both assemblages were amplified using the 4E1-HP and 5C1-P21 assays. As shown in Figure 4 (panel A), both assays were able to detect *Giardia* DNA with a theoretical limit of less of one cyst (corresponding to 16 copies of the target). In extremely diluted samples, both assays appeared to be more sensitive on assemblage B (detection of 0.15 cyst or 2 copies) compared to assemblage A (detection of 0.75 cyst or 12 copies).

Since the number of cysts of each assemblage that can be found in mixed clinical samples can be disproportioned, we tested our assays with variable proportions of DNA of the two assemblages to simulate unbalanced assemblage A+B infections. The experiments were performed with calculated ratios of 6∶4, 7.5∶2.5, and 9∶1, starting from a total amount of genomic DNA equivalent to 10 cysts. As shown in Figure 4 (panel B), the two assays yielded the expected bands in all cases, indicating that the detection of a single cyst from one assemblage is possible in the presence of nine cysts of the other assemblage. Therefore the two assays could detect mixed A+B even if the less abundant assemblage constituted 10% of the total amount of *Giardia* DNA.

### Comparison of 4E1-HP and 5C1-P21 PCR assays with other genotyping methods

The 4E1-HP and 5C1-P21 assays were used to amplify a panel of 51 DNAs from human cases of giardiasis, which have been previously characterized by multi-locus genotyping (MLG) at the ß-giardin, TPI and GDH genes [16]. The results, summarized in Table 3, showed an excellent agreement between the two sets of results. A few discrepancies were, however, noted. Thus the sample G098 was typed as assemblage A by 4E1-HP and 5C1-P21 assays, but MLG identified it as mixed A+B infection, whereas sample G162 was typed as a mixed A+B infection, but as assemblage A by MLG. Similarly, the 5C1-P21 assay detected a mixed A+B infection in sample G109, which was typed as assemblage A by MLG, whereas sample G162 was typed as a mixed A+B infection, but as assemblage A by MLG (Table 3). Of note, four samples (G077, G093, G127 and G161) could not be amplified by the 5C1-P21 assay, whereas all samples were amplified by the 4E1-HP assay.

**Table 3:** Genotyping results at the 4E1 and 5C1 markers from 51 human samples from Sweden previously characterized by MLG analysis [16].

Sample	Country of infection	MLG results	4E1 marker	5C1 marker
G046	Sweden	A	A	A
G050	Sweden	A	A	A
G067	Sweden	B	B	B
G068	Ethiopia	A+B	A+B	A+B
G077	Sweden	A	A	Neg
G093	Sweden	B	B	Neg
G094	Sweden	B	B	B
G098	India	A+B	A*	A*
G099	Egypt	A	A	A
G104	India	A	A	A
G109	India	A	A	A+B*
G110	India	A+B	A+B	A+B
G125	Gambia	A	A	A
G127	India	B	B	Neg
G129	Cuba	A	A	A
G130	Sweden	A	A	A
G135	Sweden	A	A	A
G140	India	A+B	A+B	A+B
G143	Sweden	B	B	B
G144	Sweden	B	B	B
G148	Eritrea	B	B	B
G149	India	B	B	B
G150	Egypt	A	A	A
G151	India	B	B	B
G153	Sweden	A	A	A
G155	Ghana	A	A	A
G157	Brazil	A	A	A
G161	Sweden	B	B	Neg*
G162	Sweden	A	A+B*	A+B*
G163	Eritrea	B	B	B
G168	Sweden	B	B	B
G169	Somalia	B	B	B
G170	Sweden	B	B	B
G171	Pakistan	B	B	B
G174	Ecuador	A	A	A
G177	Sweden	A	A	A
G185	Sweden	A	A	A
G188	Tanzania	B	B	B
G192	Sweden	B	B	B
G193	Egypt	B	B	B
G194	Sweden	A	A	A
G195	Bangladesh	B	B	B
G196	Kenya	B	B	B
G198	India	B	B	B
G202	Sweden	B	B	B
G203	India	B	B	B
G207	India	A+B	A+B	A+B
G208	Sweden	B	B	B
G210	Brazil	A	A	A
G211	Sweden	A	A	A
G212	Brazil	B	B	B

* Asterisks indicate results not in agreement with those obtained by MLG analysis.

Importantly, this experiment showed that the 4E1-HP and 5C1-P21 assays also detect the A2 and A3 genotypes of assemblage A, which differ from the A1 genotype used for primer design, and which are by far the most common genotypes found in humans.

## Discussion

Human giardiasis is caused by two distinct genetic groups of *Giardia duodenalis*, known as assemblages A and B, which are likely to represent distinct species [4], [10], [18]. Both assemblages are found associated with human infection globally, and have been also detected in various animals [19].

At present, various molecular methods are available to distinguish these assemblages, mainly by nested PCR followed by RFLP or DNA sequencing, or by real-time PCR [19]. The majority of these assays are based on the amplification of a gene fragment with primers that bind to DNA sequences that are conserved in the two assemblages (or conserved in all *G. duodenalis* assemblages or in *Giardia* species). Molecular typing techniques have been extensively used to study the complex epidemiology of giardiasis, including controversial aspects like the extent of zoonotic transmission, the occurrence of mixed infection in humans, the potential for genetic exchanges between parasite isolates, and the correlation between clinical symptoms and the type of assemblage [20]. In the course of these studies, it has been observed that a multi-locus typing scheme is needed to address those issues [5], [15], [16].

In this work, we have developed six novel assays that detect and discriminate *G. duodenalis* assemblages A and B using a conventional PCR procedure. Based on fixed differences at these loci between the two assemblages, specific primers were designed to generate amplicons of diagnostic size for each assemblage at each locus. The loci targeted by these assays are highly specific for *G. duodenalis* as no significant homology with DNA sequences of other organisms were detected by a BLAST search of the GenBank database (update date: 2012/03/15). Therefore, these assays represent reliable tools to identify *G. duodenalis* assemblages in human samples and can be used in combination to perform a multi-locus genotype (MLG) analysis by means of a simple PCR protocol that requires only cheap equipments. We further showed that two of the six assays (4E1-HP and 5C1-P21) are particularly well suited to allow for an effective diagnosis of the parasite in human stools with a simultaneous identification of the assemblage. We demonstrated that these two assays work efficiently in the same reaction tube, producing results that are easy to interpret (Figure 2), are highly sensitive, detecting the DNA equivalent of a single cyst (Figure 4), and appropriate to detect mixed assemblage A+B infections even if the ratio between the assemblages is strongly unbalanced (Figure 4). The two targeted loci correspond to single copy genes that are located on different chromosomes in the WB genome, namely chromosome 4 for 4E1-HP and chromosome 1 for 5C1-P21 , and these chromosomal regions are syntenic in assemblage B (data not shown). The robustness of the 4E1-HP and 5C1-P21 assays was confirmed by testing a panel of 51 human isolates from Sweden to compare the rate of amplification and the genotyping results with those obtained using MLG analysis in the original publication [16]. The excellent agreement between the two sets of results (Table 3) indicates that the two PCR assays have sensitivity and accuracy comparable with the most commonly used genotyping tools.

We therefore believe that these assays will find application in studies aimed at understanding some intriguing aspects of giardiasis, the most important of which are briefly discussed below.

The occurrence of mixed A+B infections in human cases of giardiasis appears to be more common than previously believed [21]–[22]. Thus, the proper detection of both assemblages is an important aspect for molecular epidemiology studies and for routine screening in clinical settings. The reliable detection of cases of mixed infections is influenced by several factors, including the proportion of each assemblage in the specimen and the extent of preferential amplification of one assemblage over the other. The assays presented in this work can detect the least abundant assemblage even when the other assemblage is 9 times more represented in the sample, at least using deliberately mixed genomic DNAs from axenic cultures (Figure 4). Moreover, the assemblage-specific primers do not compete for the same template, as they bind to different portions of the genes, and this should help to prevent the amplification of the most abundant template when more than one template is present. It is interesting to note that the differences among the various tests, except for false negatives, always involve samples where at least one of the assay indicates a mixed A+B infection. This fact suggests that assays with different sensitivity for one of the two assemblages not always detect correctly mixed infections. In this respect, the 4E1-HP and 5C1-P21 PCR have a proved reliability also in case of strongly unbalanced templates and demonstrated to be sensitive enough to detect mixed assemblages in the presence of a few copies of the *Giardia* genomes. Although these novel assays may be less sensitive than nested PCR in absolute terms, they are better suited to identify mixed infections and, along with previously published procedures [21]–[23], will consent to address this important aspect in future studies.

Understanding the relative contribution of the parasite's genetic variability and host factors in the establishment of clinical giardiasis has been the subject of a number of studies in both developed and developing countries [20]. So far, these studies have reached controversial conclusions. Assemblage A was associated with diarrhea (and other symptoms) in studies in India, Spain and Turkey, whereas an association with assemblage B was reported in Malaysia, The Netherlands and Ethiopia. No association with either assemblage was found in Cuba, The United Kingdom, Brazil and Albania. Whereas those conflicting results can be explained by differences in the study design, in the population considered (adults versus children), and in the definition of symptoms, is presently unknown. However, since generic PCR assays have been used in those studies, the contribution of mixed infections has not been taken into account.

Albeit the assays have been designed and tested on *G. duodenalis* isolates from humans, they can be used to detect assemblages A and B in animal samples. It is important to recall that mixed infections involving both host-specific and zoonotic assemblages of *G. duodenalis* have been frequently identified in fecal samples from livestock and domestic animals [23]–[24]. We noted that the primer pairs used in our assays do not match with the homologous sequences of the assemblage E genome [17], at least as inferred by an *in silico* analysis (data not shown). Therefore, it should be possible to use these assays to detect assemblages A and B in livestock, albeit a direct testing will be required to rule out unspecific reactions.

In conclusion, two PCR methods are proposed as valuable tools for the molecular diagnosis of human infections caused by *Giardia duodenalis*. Since these assays do not require particular expertise or expensive instruments, they can be used in laboratories with basic molecular equipments.

## Supporting Information

Table S1 **List of the clones from the** ***G. duodenalis*** **assemblage B library and the corresponding genes annotated in GiardiaDB.**
(DOC)

Table S2 **Alignments between cloned sequences (assemblage B) and the homologous sequences of** ***G. duodenalis*** **WB (assemblage A) showing the positions of the assemblage-specific primers.**
(DOC)
