# Supplementary material for: Detection of Giardia duodenalis Assemblages A and B in Human Feces by Simple, Assemblage-Specific PCR Assays
Source: PLoS Negl Trop Dis. 2012 Aug 28;6(8):e1776. doi: 10.1371/journal.pntd.0001776 (PMC3429382; doi:10.1371/journal.pntd.0001776)
Supplement: Table S1 — List of the clones from the G. duodenalis assemblage B library and the corresponding genes annotated in GiardiaDB. (DOC) [file pntd.0001776.s001.doc]

Table S1. List of the clones from the *G. duodenalis* assemblage B library and the corresponding genes annotated in GiardiaDB. The clones used to develop the PCR assays are in bold type.

| Clone | Insert (bp) | Gene ID in GiardiaDB  WB/GS | Homolog in strain P15 (assembl. E) | Annotation in GiardiaDB | Identity %  WB/GS |
| --- | --- | --- | --- | --- | --- |
| **1A3** | 882 | GL50803_114161/None | GLP15_4020 | High cystein protein | 75% |
| 1B2 | 456 | GL50803_34141/GL50581_1153 | GLP15_3864 | Nin one binding protein-like protein | 78% |
| 1D6 | 313 | GL50803_114121/GL50581_3335^*^ | GLP15_2437 | VSP | 54% |
| 1E2 | 358 | GL50803_16226/GL50581_537 | GLP15_3978 | Hypotetical protein | 87% |
| 2D4 | 305 | GL50803_40067/GL50581_2772 | GLP15_3843 | Hypotetical protein | 81% |
| 2F5 | 242 | GL50803_16122/GL50581_598 | GLP15_3278 | Kinase, NEK | 75% |
| 2F6 | 532 | GL50803_3417/GL50581_2847 | GLP15_3684 | Preimplantation protein 3 | 81% |
| 3A6 | 343 | GL50803_16321/GL50581_32 | GLP15_2224 | Hypotetical protein | 71% |
| 3B1 | 679 | GL50803_15497/GL50581_4166 | GLP15_5110 | Hypotetical protein | 67% |
| **3B4** | 816 | GL50803_16690/GL50581_2493 | GLP15_2187 | Hypotetical protein | 72% |
| 4A4 | 280 | GL50803_16592/GL50581_453 | GLP15_453 | ABC transporter family protein | 78% |
| 4A5 | 410 | GL50803_11897/GL50581_4088 | GLP15_2048 | Phosphatidylinositol-4-phosphate 5-kinase, putative | 75% |
| **4E1** | 336 | GL50803_13988/GL50581_3242 | GLP15_4687 | Hypotetical protein | 82% |
| **4F1** | 322 | GL50803_95908/None | GLP15_4518 | Hypotetical protein | 80% |
| 4F4 | 197 | GL50803_33592/GL50581_3534 | GLP15_1854 | Hypotetical protein | 83% |
| 4F6 | 373 | GL50803_94143/GL50581_4407 | GLP15_3217 | Hypotetical protein | 83% |
| **5A2** | 313 | GL50803_137610/GL50581_3193^*^ | GLP15_2468[[1]](#footnote-2)* | VSP | 63% |
| 5A4 | 386 | GL50803_14434/GL50581_1812 | GLP15_1528 | Protein 21.1 | 72% |
| 5B5 | 413 | GL50803_113740/GL50581_3794 | GLP15_3472 | Hypotetical protein | 73% |
| **5C1** | 350 | GL50803_15306/GL50581_725 | GLP15_2928 | Protein 21.1 | 77% |
| 5D4 | 372 | GL50803_137695/GL50581_1063 | GLP15_285 | Kinase, CMGC DIRK | 80% |
| 6A3 | 834 | GL50803_113030/GL50581_1990^*^ | GLP15_4054[[2]](#footnote-3)* | Kinase, NEK | 60% |
| 6B2 | 320 | GL50803_8389/GL50581_4142 | GLP15_1824 | P60 katanin | 80% |
| 6B5 | 278 | GL50803_14856/GL50581_145 | GLP15_362 | Signal recognition particle receptor | 76% |
| 6C3 | 317 | GL50803_16354/GL50581_2108 | GLP15_4447 | Protein 21.1 | 74% |
| 6E3 | 279 | GL50803_3564/GL50581_172 | GLP15_2346 | Hypotetical protein | 78% |
| 6F4 | 779 | GL50803_10260/GL50581_3948 | GLP15_2510 | Hypotetical protein | 81% |
| 7B4 | 770 | GL50803_136003/GL50581_2361^*^ | GLP15_19[[3]](#footnote-4)* | VSP | 77% |
| 8B6 | 314 | GL50803_137755/GL50581_2496 | GLP15_897 | Hypotetical protein | 79% |

1. ^*^ Homolog in a not syntenic region respect to WB. [↑](#footnote-ref-2)
2. ^*^ [↑](#footnote-ref-3)
3. ^*^ [↑](#footnote-ref-4)
