# Supplementary material for: Detection of Giardia duodenalis Assemblages A and B in Human Feces by Simple, Assemblage-Specific PCR Assays
Source: PLoS Negl Trop Dis. 2012 Aug 28;6(8):e1776. doi: 10.1371/journal.pntd.0001776 (PMC3429382; doi:10.1371/journal.pntd.0001776)
Supplement: Table S2 — Alignments between cloned sequences (assemblage B) and the homologous sequences of G. duodenalis WB (assemblage A) showing the positions of the assemblage-specific primers. (DOC) [file pntd.0001776.s002.doc]

Supplemental Table 2. Alignments between cloned sequences (assemblage B) and the homologous sequences of *G. duodenalis* WB (assemblage A). Single lines indicate the positions of primers for assemblage A. Double lines indicate the position of primers for assemblage B.

1A3/HCP

Clone 1A3 ------------------------AGCTCTGTAGATGGCAAAGGAATGTGTCAGTGTGAC 36

HCP 114161 AAGGGGCGCGGAGTATGCATCGCGAGCCTTGTGGCTGGCCAGGGAATGTGCCAGTG---C 717

*** *** * **** * ******** ***** *

Clone 1A3 AGTAACGTGTACCGCACAGACAAGAAGTCCGGCCAGTGCTTTGTCAAGGAGTGCTTCGGA 96

HCP 114161 AGTGATCAGTATCGCACAGACAAGAAGACCGGCCAGTGCTTTGTCAAGGAGTGCTTCGGG 777

*** * *** *************** *******************************

Clone 1A3 GCAGCGGACAGCATCCTCTCAGAGGTGTGCGACGGCGGCGGCACGTGTGATGAAAATACT 156

HCP 114161 GCGCATGCAAGCATCTCTGCAGAGGTATGCGATGGTGGTGGAACGTGTAATGAAAATACT 837

** * ****** ******* ***** ** ** ** ****** ***********

Clone 1A3 AAGAAGTGCAATTGTAATGTAGATGGTTTCCAGAGCCTTTCTGGTCAAAACGGCTGTGCA 216

HCP 114161 AAGAAGTGCGATTGTAATTC---TGGTTTCCAAAATCTTCCTGGCCAGAATGGCTGCGTG 894

********* ******** ********* * *** **** ** ** ***** *

Clone 1A3 CACACCAACTGCATCTCGTCGGACAGCAAGCTCTGCAGCGGCTTTGGCGCTTGTGAGAAG 276

HCP 114161 CACAGCAATTGTATTTCGTCGGACCAGAAGCTCTGCAGCGGCTTCGGCGCCTGCGAGAAT 954

**** *** ** ** ********* ***************** ***** ** *****

Clone 1A3 ATTGATGGTAGTTATCGATGCCTGTGTGCCAGCCACTACACCCTGGTGGAGAAGGACTGC 336

HCP 114161 AACAATGGCAATTACATGTGTCTGTGTGCTAACTATTACACCCTGGTTGGAAGGGACTGC 1014

* **** * *** ** ******** * * * *********** * * *******

Clone 1A3 GTTCCCACCAACTGCCTCAACGGCACAGTCACCTGCAACGGCGGCGGCACGTGCACCGGG 396

HCP 114161 ATCCCCACAAGGTGTCTCAAGGACGGGAAAGTCTGCAACGGCGGCGGCACGTGCTCCGGC 1074

* ***** * ** ***** * * ********************** ****

Clone 1A3 ACGGGCGCCTCTGCAAACTGTAGCTGCAGACAGGGCTGGGCCTCCCACGGGGCCCTCTGC 456

HCP 114161 GAGGGATTCTCTGCTACCTGCAGCTGCAGCCAGGGCTGGACCCTCCACGGGACCCTCTGC 1134

*** ****** * *** ******** ********* ** ******* ********

3B4/HP

Clone 3B4 TATCCTCGATACGTGGTACTCCGGCCTTTGGTACACGATTCACTTCGTCAATGGGAATGA 480

HP 16690 CATCCTCGACACAATGTATTCTGGGCTCTGGTTTGAACTTTACTTCCAGAATAATAGTGA 254

******** ** *** ** ** ** **** ** ***** *** * ***

Clone 3B4 TAAGGTCAACTGGCCGCTGCAGGCGTCTCTGCCCCGCACGTCCACTGCGTGGATAGAGTC 540

HP 16690 TGTGGTGTTGTGGCCTATACAGGCTTCTCTTCCTCGCACTTCTTCAGCTTGGATCGAGTC 314

* *** ***** * ***** ***** ** ***** ** * ** ***** *****

Clone 3B4 CGTCACCTTCCAGCATTCAAATGTCGCCGCGCACATCGACGAACGTCTCTGCGACTATGA 600

HP 16690 GGTGACGTTCCAGCATTCGAAGGTGGCAGCCCACATAGATGAACGCCTCTGCGATTACAA 374

** ** *********** ** ** ** ** ***** ** ***** ******** ** *

Clone 3B4 CTCTGTCGCCACGGCGTTCACGAGCGCTAAGGACATCCTCAGCATGAAGCACACCTTCTT 660

HP 16690 CGCTGTCAGCACCATCTTTATTCCCGCGAAAGACATCCTCAGTATGAGGCAGACCTTTTT 434

* ***** *** ** * *** ** *********** **** *** ***** **

Clone 3B4 CACGACGGAGGGACAGCGATTTGAGGATGGGAACATGTTCAATAATATAAGGGCCGATGA 720

HP 16690 CACTACGGCAAATCAGCAGTTTGCAGACAATAATATGTTCAATGATGCCGTAGCCGATGA 494

*** **** **** **** ** ** ********* ** ********

4F1/HP

Clone 4F1 ATCAAGTTGTGCACAGAGGCAGAGGTGCAACTTGTTCAGCTTAAGAAGCTGTCAGTAGAA 88

HP 95908 ATCAAGCTGTGCACAGATGCGGAGGTTCAGCTTATTCAGCTAAAGAAGCTATCGGTAGAG 11160

****** ********** ** ***** ** *** ******* ******** ** *****

Clone 4F1 CGTGACTCTCTGATCACTGAGAAGCAGCGTTTAGAGCAGGCAATCAGCCAAATCAGGCGT 148

HP 95908 CGTGAGTCCTTGGCTGCTGAGAAGCAGCGCTTAGAACAGGCAATCAACCAAATCCGGCGC 11220

***** ** ** ************* ***** ********** ******* ****

Clone 4F1 CAATCATCTGTGACGGAAACCAATCTGCGACGCTCGATTCAAAAAGAAAAGCATGCAATC 208

HP 95908 CAATCTTCTGTGACAGAGAATAACCTGCGGCGCTCAATCCAGAAGGAAAAGCACGCGATT 11280

***** ******** ** * ** ***** ***** ** ** ** ******** ** **

Clone 4F1 GCTAACGCTGAGACGCAGTGCACGGAAGCAATGTCTCGCGTGACAGCGGCAAAGGCCTCC 268

HP 95908 ACTACTGCCGAGACGCAGTGCACAGAGGCAATGTCTCGTGCGACAGCGACAAAGGCATCA 11340

*** ** ************** ** *********** * ******* ******* **

Clone 4F1 GTAAGCATTCTACAACAAAATGCAGCCCATATAGAGGACGAGACAGCTGAGCAG------ 322

HP 95908 GTAAGCGTCCTGCAGCAAAATGCAGCGTACATAGAAGACGAGGCGGCCGAGCAGGACAGG 11400

****** * ** ** *********** * ***** ****** * ** ******

5A2/VSP

Clone 5A2 -----------------GACCTGTGTAGAAGCTAGTGGGTGCAATGGAGCTACTTATGCC 43

VSP 137610 AAGTTCCTGAAGGCTGACAAGTGTGTGGATGCTAATCAATGTGACAATGGTAAGTATGCA 480

* ***** ** **** * ** * * ** *****

Clone 5A2 GATCCAACAACAAACAAATGCAAGAGCTGTGC---CACTGATATACCCGAGTGCMCTGCT 100

VSP 137610 GACCCAAAAACAGGCCAATGCAAGGCCTGCACGGACACAAGTGTCAATGAATGTGCTACG 540

** **** **** * ******** *** * *** * * ** ** ** *

Clone 5A2 TGCACGTATAGCGACAGTCTTCAGAAGCCGGTGTGCAGTAATTGTGGCAGTGGTGGAAA- 159

VSP 137610 TGTGCATACAGCGACACTCTTCAGAAGCCTGTGTGCACTGGGTGTAATAGTGGAGGAAAC 600

** * ** ******* ************ ******* * *** ***** *****

Clone 5A2 --GCTTCTAAGGATAGACTTGGATGGGACAACGACTTGCGTGGACGATGCTGGATGTACG 217

VSP 137610 CTGCTTCTTAAGGTGAACCCCGATGGGTCAGCGACGTGTGTTGCGGAGGCAGAGTGCACA 660

****** * * * ** ****** ** **** ** ** * ** ** * ** **

Clone 5A2 GATGGTAATACTCACTTTGTTATCGATGAGTCTGGGAAGAAGTGTCTTCTGTGTAATGAT 277

VSP 137610 AGTGGCAATACGCACTTCCTT---GAACAATCTCCAAAAGCTTGTGTTCCATGTGGTGAT 717

*** ***** ***** ** ** * *** ** *** *** *** ****

5C1/P21

Clone 5C1 ------GACCTAATGTTGGCAGTGATTAATAGAAATGCTTTCGACACGTTCTGTTACTTG 54

P21 15306 AACACTGACCTAATGCTAGCCGTAGTTAATAAGGATCCCTTTAGCACATTTTGTTACTTA 1620

********* * ** ** ****** ** * ** *** ** ********

Clone 5C1 CGGCAAGCACGATTGAGGAATATTCTGGGAATGACTGCGTTGATGATGGCAGCTAAGCAC 114

P21 15306 CGACAGGCACGATTAAGGAATGTCTTTGGCATGACTGCATTGATGTTGGCGGCCAAGTAC 1680

** ** ******** ****** * * ** ******** ****** **** ** *** **

Clone 5C1 GGAAATCAGCAAGCGATTGAGGACCTAATTGACGTAGAGGGCTGCATGCAGGTTGATCAG 174

P21 15306 GGAAATCAGCAGGCTATCGAATACTTAATTGATGTCGAAGGTGGCATGCAGGTTGATCAG 1740

*********** ** ** ** ** ******* ** ** ** *****************

Clone 5C1 CAGGATTGGATGGTTGGCGAAGAATCGACCAGTCTTGCTGGCAAAACCGCACTCATGTTT 234

P21 15306 CAAGATTGGATAGCTGATCCAGATTCGACCAGTCTTATTGGAAAGACCGCACTTATGTTT 1800

** ******** * ** *** ************ *** ** ******** ******

Clone 5C1 GCGGCAGAATCTGGGCACCTTTCTGCTGTAGCAAGGCTGTCCAGCATAGAGGCAGGTATG 294

P21 15306 GCGGCAGAAGCAGGATCTCTTTCTGCTGTTGCAAGACTAGCAAGTGTAGAGGCAGGGCTG 1860

********* * ** *********** ***** ** * ** ********** **

Clone 5C1 GTAGATAAGATTGGGGAGACGGCCCTCATGAAGGCTACGACGATGAATCATATAGA---- 350

P21 15306 GTAGATAAGGCCGGTGAAACGGCTCTCATGAAAGCCACCATAATGAATCATACGGATATC 1920

********* ** ** ***** ******** ** ** * ********** **
